# Calcification of coronary arteries and abdominal aorta in relation to traditional and novel risk factors of atherosclerosis in hemodialysis patients

**DOI:** 10.1186/1471-2369-14-10

**Published:** 2013-01-14

**Authors:** Przemysław Pencak, Beata Czerwieńska, Rafał Ficek, Katarzyna Wyskida, Agata Kujawa-Szewieczek, Magdalena Olszanecka-Glinianowicz, Andrzej Więcek, Jerzy Chudek

**Affiliations:** 1 Department of Radiology, Medical University Hospital SPSK-M, Katowice, Poland; 2 Department of Nephrology, Endocrinology and Metabolic Diseases, Medical University of Silesia, Katowice, Poland; 3 Health Promotion and Obesity Management Unit, Department of Pathophysiology, Medical University of Silesia, Katowice, Poland; 4 Pathophysiology Unit, Department of Pathophysiology, Medical University of Silesia, Katowice, Poland

**Keywords:** Atherosclerosis, Risk factors, Hemodialysis

## Abstract

**Background:**

Process of accelerated atherosclerosis specific for uremia increases cardiovascular risk in patients with chronic kidney disease (CKD) and may be influenced by the different structure of arteries. The study assesses the influence of traditional and novel risk factors on calcification of coronary arteries (CAC) and abdominal aorta (AAC) in hemodialysis patients (HD).

**Methods:**

CAC and AAC were assessed by CT in 104 prevalent adult HD and 14 apparently healthy subjects with normal kidney function (control group). Mineral metabolism parameters, plasma levels of FGF-23, MGP, osteoprotegerin, osteopontin, fetuin-A, CRP, IL-6 and TNF-α were measured.

**Results:**

CAC and AAC (calcification score ≥ 1) were found in 76 (73.1%) and 83 (79.8%) HD respectively, more frequent than in the control group. In 7 HD with AAC no CAC were detected. The frequency and severity of calcifications increased with age. Both CAC and AAC were more frequently detected in diabetics (OR = 17.37 and 13.00, respectively). CAC score was significantly greater in males. CAC and AAC scores were correlated significantly with pack-years of smoking and plasma osteoprotegrin levels. However the independent contribution of plasma osteoprotegerin levels was not confirmed in multiple regression analysis. Age (OR = 1.13) and hemodialysis vintage (OR = 1.14) were the independent risk factor favoring the occurrence of CAC; while age (OR = 1.20) was the only predictor of AAC occurrence in HD.

**Conclusions:**

1. AAC precedes the occurrence of CAC in HD patients. 2. The exposition to uremic milieu and systemic chronic microinflammation has more deteriorative effect on the CAC than the AAC.

## Background

Cardiovascular diseases remain the leading cause of morbidity and mortality, regardless of the significant progress of revascularization procedures and their accessibility, in patients with chronic kidney disease (CKD). In CKD patients, especially in those on dialysis therapy, the risk of cardiovascular death is particularly high, 10–20 times greater than in the general population [Foley et al. [1]]. At least in part the increased risk is related to accelerated development of atherosclerosis, that cannot be only attributed to the presence of the traditional risk factors such as age, sex, smoking, obesity, dyslipidemia, hypertension and diabetes. In 1974, Lindner et al. [2] described the phenomenon of the accelerated atherosclerosis in dialysis patients, that has not been fully explained for nearly forty years. The development of accelerated atherosclerosis and typical for CKD calcification of tunica media, labeled as Monckeberg’s sclerosis is associated with transformation of vascular smooth muscle cells (VSMCs) into osteoblast-like cells, able to synthesize bone matrix protein-2 (BMP-2), matrix Gla protein (MGP) and osteopontin [3]. Osteoblasts in vascular wall may also arise from mesenchymal cells - pericytes. It is believed that about 10-30% pericytes can be converted into bone cells [4].

The process of vascular calcification of systemic arteries probably differs in muscular and elastic vessels. High number of VSMCs in the tunica media of muscle arteries seems to predispose, whereas high content of elastic fibers in elastic large arteries may protect against Monckeberg’s calcifications development. It should be stress, that degradation of elastic fibers by matrix metalloproteinases with generation of soluble elastin peptides may stimulate VSMCs transformation into osteoblast-like cells [5]. This process of vascular calcification is similar to the process of bone formation.

Recent evidence suggests that the interaction of traditional (i.e., Framingham: age, lifestyle, diabetes, hypertension, dyslipidemia) and uremia-related, so could novel (e.g. hyperphosphataemia, high calcium x phosphorus product, hyperparathyroidism, oxidative stress, systemic inflammation, protein-energy wasting, asymmetric dimethylarginine, P-cresol, fetuin A) cardiovascular risk factors contribute in excessive and accelerated vascular calcifications in CKD patients [6]. In elderly CKD patients, traditional cardiovascular risk factors probable participate more than the novel ones in the development of vascular calcifications. Shlipak et al. showed that traditional cardiovascular risk factors had larger associations with cardiovascular mortality than novel risk factors in elderly CKD (non-dialysis) persons [7]. However, whether their role in the development of calcification in muscular and elastic vessels is similar is not yet known.

Therefore the aim of the present study was to assesses the influence of traditional and novel risk factors on calcification of coronary arteries and abdominal aorta in hemodialysis patients.

## Methods

One-hundred-four stable, prevalent hemodialysis patients (HD; 56 males and 48 females) from single HD unite (recruited from 2009 to 2011) and 14 apparently healthy subjects with normal kidney function (control group) were included into the study. Patients on HD therapy for less than 3 months, any acute illnesses within a month, cancers, liver cirrhosis and treated with cinacalcet were excluded from the study. Study protocol was approved by the Bioethics Committee of Medical University of Silesia (KNW-6501-37/I/08). All patients gave informed consent for participation in the study.

All patients from the HD group were on hemodialysis 3 times per week for 4–5 hours sessions. Bicarbonate-buffered dialysate fluid containing 2–3 mmol/L potassium, 1,25 mmol/L calcium, 0.75 mmol/L of magnesium and low-flux polysulfone or cuprofane dialysis membranes were used in all patients. HD patients characteristics including CKD causes, duration of hemodialysis therapy, Kt/V and comorbidities are given in Table 1.

**Table 1:** Demographic and clinical characteristics of study participants (mean & 95% CI)

	**Hemodialysis group**	**Control group**	***Statistical significance***
	**(N = 104)**	**(N = 14)**	
Age *(years)*	53.6 (50.3-56.9)	54.6 (45.1-64.1)	0.93
Gender *(male/female)*	56/48	6/8	0.63
Body mass index *(kg/m*^*2*^*)*	24.9 (23.3-26.6)	24.7 (23.9-25.6)	0.82
Obesity (BMI ≥ 30 kg/m^2^) (n/%)	11 / 10.6	0	0.43
Smokers (n/%)	62 / 59.6	1 / 7.1	<0.001
Smoking burden *(pack-years)*	15 (12–18)		
Family burden (n/%)	32 / 30.8	NA.	
Primary cause of CKD *(n/%)*			
*Diabetes*	11 / 10.6		
*Hypertension*	4 / 3.8		
*Nephrolithiasis*	3 / 2.9		
*Autosomal Dominant Polycystic Kidney Disease (ADPKD)*	6 / 5.8		
*Ischemic nephropathy*	5 / 4.8		
*Glomerulonephritis*	50 / 48.1		
*Urinary tract infection*	6 / 5.8		
*Amyloidosis*	3 / 2.9		
*Other or unknown*	16 / 15.4		
Time on dialysis *(months)*	45 (33–54)		
Kt/V *(per HD session)*	1.29 (1.25-1.34)		
Co-morbidity *(%)*			
*Hypertension*	87 / 84.5	0	
*Diabetes*	19 / 18.3	0	
*Coronary artery disease*	35 / 33.7	0	
*Stroke*	9 / 8.7	0	
*Past kidney transplantation*	18 / 17.3	-	
Pharmacotherapy *(n/%)*			
*Antihypertensive*	79 / 76.0		
*No of antihypertensive drugs (n)*	2.0 (1.7-2.2)		
Oral anti-diabetic*	5 / 26.3		
Insulin*	14 / 73.7		
*Antiplatelet*	52 / 50.0		
*Oral anticoagulant*	9 / 8.7		
*Statins*	18 / 17.3		
*Fibrates*	0		
*Oral phosphorous binders*	102 / 98.1		
*Carbonate calcium dose (g/day)*	3.3 (2.9-3.7)		
*Sevelamer hydrochloride (g/day)*	0		
*Alfacalcidol*	82 / 78.8		
*Alfacalcidol dose (μg/week)*	2.5 (1.1-3.8)		

* for patients with diabetes.

NA. – not available.

The control group consisted of 14 apparently healthy adults in similar age – range from 23 to 85 years (6 males and 8 females) with normal kidney function (eGFR-MDRD > 90 ml/min/1.73 m^2^) and without significant albuminuria.

The study protocol assumed anthropometric measurements, blood sampling performed during the morning before computed tomography (CT) scanning after overnight fast. In addition monthly, routinely measured parameters (complete blood count, serum concentrations of albumin, urea, calcium, phosphorous, intact parathyroid hormone, sodium, potassium, total, LDL and HDL cholesterol and triglycerides) were obtained before mid-week HD session. Plasma samples for estimation of CRP, IL-6, TNF-α, fetuin-A, osteoprotegerin, osteopontin, osteokalcin, MGP, intact fibroblast growth factor 23 (FGF-23) and 25(OH)D_3_ were stored frozen at −70°C till assessment.

### Measurement calcification score with multislice spiral computed tomography (MSCT)

Measurement of coronary arteries (CAC) and abdominal aorta (AAC) calcifications was performed with 64-row CT scanner Aquilion 64 (Toshiba Medical Systems Corporation) equipped with an integrated ECG monitor and appropriate software for testing and evaluation of calcium Agatston score.

Scanning was done in sequential 3 mm thick layers (for aorta starting scanning 3 cm proximal to the bifurcation of the aorta). After the test, the images were transferred to a dedicated workstation VITREA 2 (Vital Images, Inc.) equipped with program to calculate Agatston score. Calculations were done automatically after determination by the radiologist the calcification in the arterial wall in appropriate scans. The results were read-out of the workstation monitor.

### Laboratory analyses

Routine laboratory measurements were performed in hospital laboratory (Synchron Cx-9, Beckman Coulter Inc., Fullerton, CA, U.S.). Serum high sensitivity CRP was measured by nephelometry (Siemens Healthcare Diagnostics, Deerfield, IL, U.S.) with a lower limit of sensitivity of 0.2 mg/l.

Plasma concentrations of IL-6 (R&D System, Minnesota, MN, U.S.), TNF-α (R&D System, Minnesota, MN, U.S.), MGP (Biomedica, Wien, Austria), fetuin-A and osteopontin (DRG, Mountainside, NJ, U.S.), osteoprotegerin (Biovendor, Modřice, Czech Republic), osteocalcin (Quidel, San Diego, CA, U.S.), 25-OH-D_3_ (Immundiagnostik, Bensheim, Austria), and FGF-23 (Immuntopics, San Clemente, CA, U.S.) were determined by ELISA.

### Data analysis

Arterial hypertension was defined as the predialysis blood pressure values ≥140/90 mmHg or antihypertensive medication. Dyslipidemia was defined as the concentration of total cholesterol ≥ 5.0 mmol/L or LDL-cholesterol ≥ 3.0 mmol/L or low HDL-cholesterol (< 1.0 mmol/L for men and < 1.2 mmol/L for women) or triglycerides ≥ 1.7 mmol/L or lipid lowering therapy. Obesity was diagnosed according WHO criteria.

On the basis of CAC Agatston score we classified calcifications in patients according to Rumberger et al. criteria as: mild (1–10), moderate (11–100), large (101–400) and massive (> 400) [8]. The severity of AAC were scored according own classification, based on an exponential scale: mild (1–100), moderate (101–1000) and large (> 1000).

HD patients were divided into 3 subgroups: with calcification in both locations (CAC and AAC), with isolated aorta calcifications (AAC only) and without calcifications.

### Statistical analysis

Statistical analysis was performed with STATISTICA 10.0 PL Stat Soft Corporation software (http://www.statsoft.com). Results are given as mean values with 95% confidence intervals (95% CI). For comparison of groups, we used the χ^2^ test and χ^2^ test for trend (qualitative variables) and ANOVA, followed by Tukey’s test or U-Mann–Whitney test, as appropriate (quantitative variables). Correlation coefficient was calculated according to Spearman. Univariate and multivariate backward stepwise logistic regression analysis were performed including factors potentially favoring or preventing the calcification development, including age, hemodialysis vintage, gender, hypertension, diabetes, current smoking or pack-years exposition, levels of phosphorous, calcium, PTH, 25(OH)D_3_, osteoprotegerin, MGP, CRP, IL-6, TNF-α. All variables were tested for the presence of multi co-linearity, which was assessed with the variance inflation factor and the conditional index. p < 0.05 was considered as statistically significant.

## Results

Characteristics of study groups is given in Table 1. CAC (calcification score ≥ 1) were found in 76 HD patients (73.1%), and 5 in the control group (35.7%) - p <0.01 (with Yates correction). Among HD patients 6 had mild (5.8%), 10 moderate (9.6%), 12 large (13.5%) and 48 massive (46.1%) CAC.

**Figure 1:**
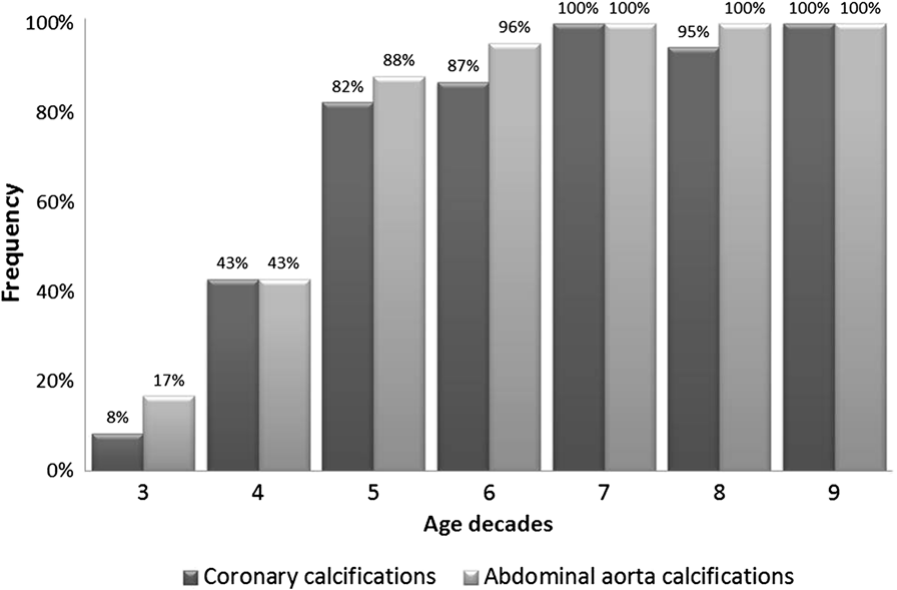
Frequency of coronary and abdominal aorta calcifications.

Within the abdominal aorta calcifications were detected in 83 HD patients (79.8%) and 6 in the control group (42.9%) - p <0.01 (with Yates correction). Among HD patients mild AAC were found in 7 subjects (6.7%), moderate in 19 (18.3%) and severe in 57 (54.8%). The frequency of calcifications increased with age (Figure 1).

The severity of CAC and AAC was significantly greater in HD patients than in the control group (Table 2). Among HD patients, AAC was detected from age of 25 years, while CAC from age of 28 years. CAC score (CACS) was significantly higher in males than in females [1196 (720–1672) vs. 644 (324–964), p = 0.001], while AAC score was similar [2971 (1968–3974) vs. 2544 (1558–3530), p = 0.49; respectively]. Calcifications in both locations were detected in each patient diagnosed with diabetes [OR = 17.37 (1.01-298.56), p = 0.049 and 13.00 (0.75-224.58, p = 0.08) for CAC and AAC respectively], and were more severe than in not diabetics [1464 (704–2224) vs. 825 (502–1147), p = 0.001 and 4987 (2861–7112) vs. 2279 (1591–2967), p < 0.001; respectively]. Diabetic patients were significantly older [64 (58–70) vs. 51 (48–59) years, p = 0.003] but not HD vintage was similar. There was no association between occurrence of calcifications and smoking or dyslipidemia. Among smokers there were correlations between pack-years and CAC as well as AAC scores (R = 0.37, p = 0.002 and R = 0.36, p = 0.002; respectively).

**Table 2:** Calcification scores and biochemical characteristics of study groups (mean & 95% CI)

	**Hemodialysis group**	**Control group**	***Statistical significance***
	**(N = 104)**	**(N = 14)**	
CACS	941 (645–1238)	182 (0–405)	<0.001
AACS	2773 (2078–3470)	431 (0–945)	<0.001
Hematocrit *(%)*	33.6 (32.8-34.5)	40.9 (38.5-43.3)	<0.001
Hemoglobin *(g/dL)*	11.1 (10.8-11.4)	14.0 (13.2-14.8)	<0.001
Albumin *(g/dL)*	3.87 (3.76-3.97)	4.06 (3.82-4.31)	0.26
Creatinine (μmol/L)	NA	77 (66–88)	
Total cholesterol *(mmol/L)*	4.63 (4.37-4.88)	5.43 (4.88-5.97)	0.02
LDL cholesterol *(mmol/L)*	2.61 (2.39-2.83)	3.17 (2.71-3.64)	0.04
HDL cholesterol *(mmol/L)*	1.14 (1.06-1.23)	1.54 (1.26-1.83)	0.002
Triglycerides *(mmol/L)*	1.94 (1.67-2.22)	1.44 (0.72-2.15)	0.02
Dyslipidemia *(n /%)*	75 / 72.1	11 / 78.6	0.85
Calcium *(mmol/L)*	2.17 (2.14-2.20)	2.26 (2.22-2.31)	0.02
Phosphorous *(mmol/L)*	1.88 (1.74-2.03)	1.04 (0.95-1.13)	<0.001
Parathyroid hormone *(pg/mL)*	409 (313–506)	50 (40–61)	<0.001
25(OH)D_3_*(ng/mL)*	22.9 (17.7-28.1)	28.0 (16.5-39.4)	0.34
iFGF-23 *(ng/mL)*	662.9 (464.3-861.4)	6.3 (5.6-6.9)	<0.001
Osteocalcin *(pmol/L)*	39.2 (31.1-47.3)	5.4 (3.9-6.8)	<0.001
Osteopontin *(ng/mL)*	103.2 (93.5-112.8)	27.8 (23.8-31.8)	<0.001
Osteoprotegerin *(pmol/L)*	14.7 (12.0-17.3)	5.0 (3.8-6.1)	<0.001
Matrix GLA protein *(nmol/L)*	11.1 (10.2-11.9)	13.6 (12.2-14.9)	0.01
Fetuin-A *(g/L)*	0.63 (0.55-0.70)	0.70 (0.56-0.84)	0.13
CRP *(mg/L)*	12.6 (9.2-16.1)	1.2 (0.8-1.6)	<0.001
IL-6 *(pg/mL)*	7.3 (5.4-9.1)	2.6 (1.3-3.8)	<0.001
TNF-α *(pg/mL)*	10.6 (8.8-12.4)	1.8 (1.0-2.5)	<0.001

NA – not available.

All but two HD patients, with CAC detected, had AAC. However, in 7 patients with AAC no CAC was detected (kappa coefficient 0.798, 95% CI 0.708-0.870). The correlation between CAC and AAC scores was slightly stronger for control group (R = 0.70, p <0.001 in HD and R = 0.78, p = 0.001 in controls).

### Characteristics of patients with calcifications

Patients with detectable both CAC and AAC were 29 years older and had longer history of HD therapy than those without calcifications (Table 3). This group included all diabetics, all but 2 with CAC, the highest percentage of patients with hypertension and low PTH level (<100 pg/mL). Moreover, it was characterized by the lowest 25(OH)D_3_ and highest osteoprotegerin and CRP plasma levels.

**Table 3:** Comparison of subgroups according localization of CAC and AAC (mean & 95% CI)

	**AAC and CAC**	**Isolated AAC**	**No calcifications**	***ANOVA***
	**(N = 76)**	**(N = 7)**	**(N = 19)**	
Age *(years)*	60 (57–63)	48 (33–63)	31 (27–34)	<0.001
Gender *(male/female)*	41/35	4/3	10/9	0.95
Smoking (n /%)	45 / 59.2	5 / 71.4	12 / 63.2	0.67
Diabetes (n /%)	19 / 25.0	0	0	0.001
Hypertension (n /%)	68 / 89.5	6 / 85.7	13 / 68.4	0.02
Coronary artery disease (n /%)	33 / 42.3	2 / 28.6	0	<0.001
Time on dialysis *(months)*	45 (32–59)	22 (10–34)	36 (15–56)	0.48
Kt/V	1.30 (1.25-1.35)	1.24 (0.93-1.54)	1.31 (1.20-1.42)	0.75
Body mass index *(kg/m*^*2*^*)*	25.2 (24.3-26.2)	23.0 (18.9-27.2)	23.1 (20.9-25.3)	0.09
Hemoglobin *(g/dL)*	11.1 (10.7-11.4)	11.4 (10.4-12.5)	11.0 (10.2-11.8)	0.80
Albumin *(g/dL)*	3.82 (3.70-3.94)	3.75 (3.21-4.30)	4.16 (3.91-4.29)	0.08
Total cholesterol *(mmol/L)*	4.78 (4.48-5.08)	4.26 (3.90-4.61)	4.08 (3.40-4.77)	0.09
LDL cholesterol *(mmol/L)*	2.75 (2.50-3.01)	2.44 (1.91-2.98)	2.19 (1.63-2.75)	0.12
HDL cholesterol *(mmol/L)*	1.16 (1.05-1.27)	1.05 (0.89-1.21)	1.11 (0.93-1.29)	0.76
Triglycerides *(mmol/L)*	2.00 (1.67-2.32)	1.75 (0.94-2.56)	1.52 (1.27-1.77)	0.32
Dyslipidemia *(n /%)*	57 / 75.0	3 / 42.9	13 / 68.4	0.37
Calcium *(mmol/L)*	2.17 (2.13-2.21)	2.22 (2.09-2.36)	2.16 (2.07-2.25)	0.68
Phosphorous *(mmol/L)*	1.78 (1.62-1.94)	1.94 (1.27-2.61)	2.16 (1.78-2.54)	0.12
Phosphorous >1.77 mmo/L (*n /%*)	40 / 52.6	3 /42.9	14 /73.7	0.14
CaxP (mmol^2^/L^2^)	3.84 (3.49-4.18)	4.31 (2.26-5.77)	4.64 (3.84-5.43)	0.12
PTH *(pg/mL)*	364 (263–464)	310 (138–481)	577 (225–928)	0.22
PTH <100 pg/mL *(n /%)*	20 / 25.6	0	2 / 10.5	0.06
25(OH)D_3_*(ng/mL)*	19.2 (14.2-24.2)	24.2 (12.7-34.9)	29.2 (17.4-40.9)	0.04
iFGF-23 *(ng/mL)*	638.0 (379.1-697.0)	955.9 (0–2524.2)	698.8 (363.5-1034.0)	0.32
Osteocalcin *(pmol/L)*	36.4 (26.7-46.0)	32.3 (14.0-50.6)	47.7 (25.7-69.7)	0.50
Osteopontin *(ng/mL)*	98.1 (86.8-109.4)	124.4 (104.9-143.9)	98.5 (80.3-116.7)	0.31
Osteoprotegerin *(pmol/L)*	17.5 (14.1-20.9)	12.0 (3.7-20.3)	6.4 (4.3-8.5)	0.003
Matrix GLA protein *(nmol/L)*	11.5 (10.5-12.5)	9.1 (4.0-14.2)	9.8 (7.9-11.8)	0.13
Fetuin-A *(g/L)*	0.60 (0.50-0.69)	0.72 (0.39-1.05)	0.68 (0.51-0.85)	0.54
CRP *(mg/L)*	12.9 (9.3-16.6)	10.6 (7.2-18.1)	8.2 (2.4-14.1)	0.03
IL-6 *(pg/mL)*	7.5 (5.3-9.6)	6.8 (4.2-8.4)	8.1 (1.0-15.3)	0.68
TNF-α *(pg/mL)*	11.1 (8.9-13.3)	6.8 (2.5-9.7)	11.4 (7.1-15.7)	0.22

The subgroup of patients with isolated AAC was younger (average of 12 years) than the subgroup with calcifications in both locations examined. This subgroup had also markedly higher plasma osteoprotegerin level (Tab. 3).

Serum phosphorous level and CaxP product were comparable in all HD study subgroups.

### Univariate correlations

In patients with CAC, CACS was significantly associated with the HD vintage (R = 0.27, p = 0.02), pack-years of smoking (R = 0.37, p = 0.002), duration of hypertension therapy (R = 0.28, p = 0.03), plasma levels of osteoprotegerin (R = 0.32, p = 0.01) and MGP (R = 0.305, p = 0.03). The severity of AAC was only related to age (R = 0.44, p < 0.001), plasma level of osteoprotegerin (R = 0.32, p = 0.003) and pack-years of smoking (R = 0.36, p = 0.002).

There were significant inverse correlation between age and serum phosphorous level (R = − 0.41, p < 0.001), CaxP product (R = −0.40, p < 0.001), PTH (R = −0.25, p = 0.01).

Moreover, there was a strong correlation between age and plasma osteoprotegerin level (R = 0.60, p < 0.001) as well as IL-6 (R = 0.28, p = 0.03).

### Multiple logistic regression analysis

The multiple logistic regression analysis showed that only age (per year) [OR = 1.13 (1.07-1.18), p < 0.001] and time on HD therapy (per year) [OR = 1.14 (1.00-1.34), p = 0.04] were independent risk factors favoring the occurrence of CAC; while age [OR = 1.20 (1.11-1.30), p < 0.001] was the only predictor of the AAC occurrence in HD patients.

## Discussion

The results of our study demonstrate that AAC precedes the occurrence of CAC and reveal differences in factors favoring their development. Age and HD vintage was equally (OR = 1.13 and 1.14) important for the occurrence of CAC, while only age was a major (OR = 1.20) predictor of the occurrence of AAC. Other traditional risk factors of atherosclerosis, correlated with CAC in our study, included diabetes and duration of hypertension therapy. All but one patients with obesity had CAC, but the limited number of obese patients precluded any statistical significance. We also failed to prove the association between dyslipidemia and CAC and AAC, probably as lipid disorders in CKD certainly differs from those prior to HD therapy and reflects only the current status and overlapping treatment. We did not analyze the association between physical activity and calcification status as the group had highly heterogeneous age. Moreover, we did not analyze the long-term nutritional habits due to the lack of research tools.

The prevalence of CAC (73.1%) is similar as previously reported - 65-90% [9-12]. However, it was lower than reported by Goodman et al. in young adults with 2-time longer dialysis vintage (7 ± 6 years) [13]. This pointing out the important influence of long-term vascular wall exposition on *uremic milieu*, later confirmed by Raggi et al. [10]. Additionally, in our study the frequency of calcifications increased with age especially during the third and fourth decade.

Among the most well-known and studied uremic abnormalities associated with development of vascular calcifications were mineral disturbances: hyperphosphatemia, high CaxP product, secondary hyperparathyroidism and adynamic bone disease. However, we did not find any difference in serum phosphorous and CaxP product, measured in a single sample, between patients with and without calcifications and the relationships between serum phosphorous and severity of calcifications in both localizations studied. This is not the only study that failed to demonstrate such a relation [12,14,15]. However, this observation does not exclude the driving force of phosphorous in the development of vascular calcifications as the subgroup with calcifications was significantly longer exposed to phosphorous disturbances (longer hemodialysis vintage). The lack of lower serum phosphorous concentration in significantly younger subgroup yet without vascular calcification can be explained by the more frequent non-adherence of younger patients to phosphorous binding drugs and diet recommendation [16]. Another possible explanation is time-dependent variation of serum phosphorous related to variability of dietary phosphate consumption, phosphate binder doses and duration of hemodialysis sessions. Recently Cianciolo et al. tried to overcome this problem calculating the period of exposure to phosphorous over 5.5 mg/dl and CaxP product over 55 mg^2^/dl^2^ recorded during last 24 months [11]. In this study the independent contribution in multivariate regression was only shown for nondiabetic HD patients. This cited data and results of our study may suggest that during the last decade an important improvement of the mineral disturbances management was obtained, inter alia by dose reduction of calcium phosphorous binders and active vitamin D metabolites, introduction of sevelamer hydrochloride and cinacalcet, recently. The progress in secondary hyperparathyroidism management was associated with increased frequency of adynamic bone disease (ABD), and the risk for vascular calcification development [17]. In our study in subgroup with vascular calcification one of the four patients had low PTH level that characterize patients with ABD.

Currently it is suggested that FGF-23 is a very sensitive marker of phosphorous disturbances. Additionally, an association between plasma FGF-23 levels and CACS or AAC were recently reported in studies assessed relatively small groups of hemodialysis patients with extremely high FGF-23 levels [18,19]. These interesting data were not confirmed by the results of lager study performed by Cianciolo et al. [11] and presented in our paper. It should be emphasized, that no FGF-23 vascular toxicity has been demonstrated yet.

There are several line of evidence showing that CAC are associated with systemic inflammation [12,14,15], as the component of the malnutrition-inflammation-atherosclerosis syndrome. Atherosclerosis frequently coexists with systemic inflammation especially in hemodialysis diabetic patients [20]. Experimental studies demonstrated that TNF-α in dose-dependent manner induce differentiation of VSMCs into osteoblast-like cells and stimulate mineral deposition [21]. We have also shown that patients with CAC and AAC had significantly higher serum CRP levels but not IL-6 and TNF-α. However, there was no correlation between circulating inflammatory markers levels and calcification scores. Time-dependent high variability is a potential cause of the conflicting results.

The process of vascular calcifications has natural inhibitors, such as MGP, osteopontin, osteoprotegerin, fetuin-A and pyrophosphates [5]. However, in our study only plasma level of osteoprotegerin was significantly increased in patient with than without calcification and was related to their severity. The association between CAC and higher circulating osteoprotegerin concentration has been previously revealed by Barreto et al. in HD patients. The authors suggested that it represents an incomplete self-defensive response to the progression of atherosclerosis [22]. Similarly, it has also been shown that increased serum osteoprotegerin levels correlates with AACS [23]. Moreover, osteoprotegerin levels were associated with progression of CACS in HD patients [24]. The lack of independent contribution to the occurrence of CAC and AAC in our study suggest that circulating osteoprotegerin level is rather a marker of vascular pathology in HD patients.

In our study the assessment of AAC was performed with own method developed, similar to the evaluation performed in the CAC. While, in the earlier studies the assessment was based on the index coverage perimeter of the aortic wall [14]. We consider, the method used in our study is more precise. In consequence we detected higher prevalence of abdominal aorta calcifications than previously reported. Okuno et al. in abdominal X-ray imaging shown AAC in 56.6% of hemodialysis patients [25]. In the large multicenter CORD study including 933 adult hemodialysis patients [26], the prevalence of AAC was similar to those reported by us (81 vs. 79.9%). In this study the occurrence of AAC were related to age and dialysis vintage. While, we were unable to prove the effect of dialysis vintage on the occurrence and severity of AAC. On the other hand we showed the association between diabetes coexistence or smoking and the severity of calcifications, as was previously observed in the general population [27].

Our study shows not only some differences in factors associated with the occurrence of vascular calcification in elastic (abdominal aorta) and muscular (coronary) arteries but also demonstrate that AAC in HD patients develops usually in younger age than CAC does. This statement is also supported by the identification of a group of 7 patients with isolated AAC. This group of patients was by more than a decade younger, and additionally had remarkably shorter hemodialysis vintage than those with calcifications in both locations. This may suggest either the different effect of age or dialysis vintage on the development of vascular calcification in both locations. Though, the existence of positive correlation between hemodialysis vintage and CAC but not AAC suggest more deteriorative effect of uremic milieu on the pathogenesis of coronary vessel pathology. While, the development of AAC seems to be more dependent on traditional cardiovascular risk factors (i.e. age). This interesting observation necessitate further studies.

Our study has some limitations. The main one is the cross sectional study design and the heterogeneity of the study group in aspect of CKD causes, age and dialysis vintage. We cannot exclude that the strong impact of age does not mask weaker effects of mineral disturbances. Additionally, we did not assessed the uncarboxylated MGP fraction, but total MGP, that limits conclusions of their role for vascular calcification development. Moreover we did not analyzed the impact of prior to CKD diagnosis factors related to lifestyle, except smoking.

## Conclusion

1. AAC precedes the occurrence of CAC in HD patients. 2. The exposition to uremic milieu and microinflammation has more deteriorative effect on the CAC than the AAC.

## Abbreviations

CAC: Calcification of coronary arteries; ACC: Calcification of abdominal aorta; HD: Hemodialysis; VSMCs: Vascular smooth muscle cells; BMP-2: Bone matrix protein-2; MGP: Matrix Gla protein; CT: Computed tomography; MSCT: Multislice spiral computed tomography; CACS: Coronary arteries calcification score; AACS: Abdominal aorta calcification score; ABD: Adynamic bone disease.

## Competing interests

The authors have declared that no competing interest exists.

## Authors’ contributions

PP – carried out CT analysis, participated in the design of the study and drafted the manuscript; BC – participated in the patients recruitment and drafted the manuscript; RF - participated in the data collecting and drafted the manuscript; KW - participated in the data collecting and drafted the manuscript; AK-Sz - participated in the data collecting and drafted the manuscript; MO-G - participated in the design of the study and drafted the manuscript; AW - participated in the design of the study and drafted the manuscript; JCh - participated in the design of the study, funds collecting, performed the statistical analysis and drafted the manuscript. All authors read and approved the final manuscript.

## Pre-publication history

The pre-publication history for this paper can be accessed here:

http://www.biomedcentral.com/1471-2369/14/10/prepub
